# Biographical Sketch of Samuel Henry Dickson, M. D., LL. D.

**Published:** 1857-02

**Authors:** 


*Biographical Sketch of* SAMUEL HENRY DICKSON, M. D., LL.D., Professor of the Theory and Practice of Medicine in the Medical College of the State of South Carolina. By THE EDITOR OF THE CHARLESTON MEDICAL JOURNAL & REVIEW.

The subject of this Sketch was born in September, 1798. His early education was obtained in his native city, Charleston, South Carolina. He went to Yale College at the age of 13, and joined the Sophomore Class, graduating in 1814 under the Presidency of Dr. Dwight. Returning home, he commenced at once the study of Medicine in the office of Dr. Philip Gendron Prioleau; in 1817 he began to practice during the prevalence of Yellow Fever: in the two succeeding winters he attended, in the University of Pennsylvania, the Lectures of Wistar, Physick, Dorsey, Chapman, and others; and received in 1819 the diploma of M. D. He entered at once into business, assisting Dr. Charles H. Glover through the pestilential summer in the Marine and Yellow Fever Hospitals; and has continued ever since to be as fully employed in the profession as he has desired.

Ambitious of distinction and usefulness, he projected, with Drs. Ramsay and Frost, the establishment of a Medical College, and, while laboring to carry the enterprise into effect, gave lectures gratuitously on Physiology, as Dr. Ramsay did on Surgery The College went into operation in 1824, Dr. D. being elected without opposition to the Chair of Institutes and Practice. Dissentions occurring with the Medical Society of South Carolina—the nominal Trustees of the School—he resigned his seat in 1832, but resumed it the next year under a new charter obtained by his colleagues from the Legislature.

In 1847 he accepted the unanimous invitation of the Trustees and Faculty of the New York University to fill the Chair of Practice in that Institution, made vacant by the death of Professor Revere. He lectured three winters in the city of New York with approbation and success; but in 1850 he was induced to return to the South, by the earnest wish of his friends and associates there. The resignation of the Chair of Surgery by Professor Bellinger, and the resumption of its duties by Professor Geddings, who had meanwhile filled that of Practice, left the latter open for its former occupant, who had so long held and still occupies it. The whole arrangement presents a rare, if not unparalleled instance of generous self-sacrifice.

On his arrival in Charleston, Dr. Dickson was greeted with the most cordial welcome; and he was honored by his professional friends and others with a public dinner, at which the most complimentary testimonials of esteem and regard were tendered him, which were gratefully acknowledged and reciprocated. In 1851 he presided at a similar entertainment given to the distinguished Marshall Hall by the physicians of Charleston. In that year he received from the New York University the honorary degree of LL.D., a compliment peculiarly agreeable to him, as betokening a kind remembrance of his former relations with that Institution; and a mark of distinction worthily conferred, because of his high erudition and the eminent position which he occupies in the Republic of Letters.

Devoted to his Profession, Dr. Dickson has kept up with the scientific progress of his day; he reads very much, and cnltivates literature assiduously, his style being pure Anglo-Saxon. Nothing in the domain of human intelligence escapes his vigilant eye; the amount of matter thus ingested being thoroughly digested and subjected to his prodigious powers of analysis. He has written extensively for the Journals and Reviews on every variety of topics. A small volume of his verses—printed but not published—was favorably noticed some years since in the Southern Literary Messenger; and is referred to in Duykinck's Cyclopedia. At various times and places he has delivered speeches, lectures, and addresses upon almost every subject of human interest—religious, political, social, educational, and miscellaneous. Among these we may mention the Oration before the Phi Beta Kappa Society of Yale College at the Commencement of 1842, when the Hon. J. C. Calhoun failed, from ill-health, to pronounce the Oration before the Alumni; the Annual Address to the Georgia Historical Society in 1845, at Savannah; an Address to the Agricultural Society of Greenville, So. Ca., in 1847; and the Centennial Oration before the Grand Lodge of Ancient Free Masons of So. Ca., in 1854.

To him belongs the credit of having made the first *Temperance* Address ever uttered South of Mason and Dixon's line, taking then the position he has ever held, denouncing the habitual use of *distilled* liquors as beverages, while he approves of, and favors the employment of the various products of mere fermentation. Hence he warmly advocates and takes pleasure in the success of all efforts to grow the grape in our country, and to manufacture its genial juice into wines of every character and quality.

He is the author of a large number of monographs, dispersed through the American Medical Journals; of two volumes entitled "Outlines of Lectures upon the Theory and Practice of Medicine;" of "Essays on Life, Death, Pain, Intellection, and Hygiene;" and of "Elements of Pathology and Practice." The last mentioned work, although intended by the Author chiefly as a Text Book for his classes, yet contains material from which even the mature practitioner may derive instruction. In the first part of the work—that, namely, which treats of general Pathology—the subjects of Irritation, Congestion, and Inflammation—their differences and their relations—have received the most complete as well as the most lucid exposition, of which, in our opinion, they are susceptible; and the essay on the "Types in Fever" is a model of research, analysis, and philosophical deduction.

In his private office, he has taken charge of, and educated a great number of attached pupils—about 70.

He claims to have been among the first to introduce or accept the modified humoral Pathology of the present day; among the first to abandon the ultra heroic treatment of fevers in vogue at the time of his entrance into the profession; among the first to admit and yield to the force of the evidence adduced in modern times and still accumulating in proof of the communicability and contagiousness of our Western pestilence, Yellow Fever; among the first in this country to extend the employment of stimulants and anodynes in febrile diseases.

His earlier lectures were written out fully and with great care; for the last ten or twelve years, however, they are entirely oral; he enters the lecture room without the smallest note or memorandum, and with wonderful mental arrangement, pronounces extemporaneously his discourses, which are characterised by great perspicuity, fluency, and command of language.

In the practical part of the profession he is distinguished by rapid mental action, quickness and brilliancy of perception ; taking in, as it were, at a glance, all the features of the case, and arriving promptly at a conclusion.

By reason of his profound knowledge of general Pathology, he rarely errs in diagnosis ; and we hesitate not to state—the statement being founded upon a long course of attentive observation, in which there has been abundant opportunity for verification or falsification—that in the matter of *prognosis* he approaches very near to infallibility. Nor is his knowledge in these respects limited to the practice of *Medicine* (the branch which he professes) ; for although he has always eschewed Surgery, yet he is occasionally consulted in obscure cases by his brethren who practice it on an extensive scale, and his views not only receive due consideration, but are frequently adopted and acted upon.

In medical and other polemics, he wields a Damascus blade of the keenest edge ; and wo unto him who comes under its stroke ! Master of the principles of logic, he seizes the weak point in his opponent's argument, and exposes its sophistry ; and possesses rare ingenuity in extricating himself from a false position, should he be so placed, and eluding his adversary's grasp.

In his occasional efforts at public speaking he has acquired enviable distinction, not only by the graces of his oratory, but also by the cogency, force, and point of his remarks, and by the beauty and chasteness of the language in which they are rendered.

No one can know Dr. Dickson without being convinced that not only has Nature been lavish in her gifts to him, but that they havebeen industriously and successfully cultivated. Conspicuously does he bear the stamp of excellence ; he would have excelled in the Forum as he has in the apparently humbler, but nobler sphere which he has chosen for his action.

He has of late shrunk from the burden and fatigue of general practice, and has confined himself to consultations with his professional brethren, in which he takes great interest. He has never enjoyed good health. His constitution, originally feeble, has been undermined by the severe and incessant study to which he has been devoted ; his youth was constantly threatened with Phthisis, the hereditary disposition to which was shown by occasional hæmoptysis and habitual cough ; and his advancing age has been embittered by the severest pangs of renal disease, and some obscure form of agonizing visceral neuralgia.

In closing this hasty sketch, we will only remark that Dr. Dickson's social life is characterised by the exercise of a colloquial power so remarkable as to throw a charm over all who are subjected to its influence ; and to render him a conspicuous and attractive member of every circle in which he mingles.

